# Clinical Lessons to Be Learned from Patients Developing Chronic Myeloid Leukemia While on Immunosuppressive Therapy after Solid Organ Transplantation: Yet Another Case after Orthotopic Heart Transplantation

**DOI:** 10.1155/2014/890438

**Published:** 2014-11-16

**Authors:** Christian Oberender, Lorenz Kleeberg, Nicola Nienhues, Martin Gresse, Bernd Dörken, Hanno Riess, Philipp le Coutre

**Affiliations:** ^1^Medizinische Klinik mit Schwerpunkt Hämatologie und Onkologie, Universitätsmedizin Berlin, Campus Mitte Charité, Schumannstraße 20/21, 10117 Berlin, Germany; ^2^Deutsches Herzzentrum Berlin, Abteilung für Herz-, Thorax- und Gefäßchirurgie, Augustenburger Platz 1, 13353 Berlin, Germany; ^3^Medizinische Klinik mit Schwerpunkt Hämatologie, Onkologie und Tumorimmunologie, Universitätsmedizin Berlin, Campus Virchow Charité, Augustenburger Platz 1, 13353 Berlin, Germany

## Abstract

Chronic myeloid leukemia developing after transplantation of solid organs and concomitant immunosuppression is a rare but still significant clinical phenomenon. We here describe an additional case of a 62-year-old male patient developing CML after orthotopic heart transplantation and medication with cyclosporine A, mofetil-mycophenolate, and steroids. Initial antileukemic therapy was imatinib at a standard dose and within 15 months of therapy a complete cytogenetic response was noted. In this report we discuss the clinical implications of these rare but biologically important cases.

## 1. Introduction

Secondary malignancies following solid organ transplantation (SOT) and immunosuppressive therapy are becoming an emerging issue as total transplant numbers are increasing, the overall survival of organ recipients is improving, and an increasing proportion of organ recipients is of older age, when cancer is more often diagnosed. Probably, the best studied scenario in this setting is the association of EBV and posttransplantation lymphoproliferative diseases [1]. But also squamous cell carcinomas and Kaposi sarcomas as well as specific myeloid malignancies, notably myelodysplastic syndromes and acute myeloid leukemia, have been observed more frequently in patients following solid organ transplantation [2, 3]. In addition, after the first report by Battin et al. (1976), a remarkable number of case reports describing cases of chronic myeloid leukemia (CML) following solid organ transplantation and immunosuppression were published over the past decades ([4–27], Table 1).

In CML the BCR-ABL1 fusion gene, caused by a typical t(9;22)(q34;q11) chromosomal translocation, is the pathogenic driver of the disease leading to enhanced proliferation and left shift but also is used as a target to monitor minimal residual disease by quantitative reverse-transcription quantitative polymerase chain reaction (rt-PCR) analyses [28]. On the protein level, the bcr-abl^p210^ oncoprotein has become the target structure for specific therapies by tyrosine kinase inhibitors such as imatinib, nilotinib, and/or dasatinib in the first-line setting and additionally bosutinib and ponatinib beyond initial treatment [29]. There are conflicting data whether BCR-ABL1 is the only oncogenic lesion that is needed to cause chronic phase CML or if other, yet unidentified, mutations are involved. In this regard the sporadic identification of low levels of BCR-ABL1 transcripts in healthy individuals was of note [30, 31]. The general interpretation of these findings was that in healthy individuals the occurrence of oncogenic mutations, including BCR-ABL1, may occur but is under the control of an effective immune surveillance.

Thus, CML developing in patients following SOT may be triggered by the effects of concomitant application of immunosuppressive drugs. In line with this hypothesis we previously compared by rt-PCR the BCR-ABL1 status in patients following SOT but without CML and indeed detected 5% low-level PCR positivity in these patients as compared to none in a control group without immunosuppression [21]. Finally, in a recently published article the risk to develop CML in patients after SOT was calculated to be more than 20-fold higher as in the normal population [26].

In this paper we describe another patient with chronic phase CML following orthotopic heart transplantation and immunosuppression. As in our previous article we discussed the various biological mechanisms that may contribute to CML following immunosuppression; we here provide an overview on all published cases and focus on individual aspects of the clinical management in this specific cohort [21].

## 2. Case Presentation

We here report on a caucasian male with dilated cardiomyopathy, who underwent in November 2010 as a 61-year-old patient an orthotopic bicaval heart transplantation at the Deutsches Herzzentrum Berlin following left ventricular assist device (HeartMate II) implantation in September 2009 due to cardiogenic shock. Due to impaired graft function a primary intra-aortic balloon pump was implanted. Transplantation was done in a CMV-positive recipient with a heart graft from a CMV-positive donor. Induction therapy was performed using Thymoglobulin (1.5 mg/kg). Posttransplant maintenance immunosuppressive therapy consisted of cyclosporine A (4 mg/kg), mofetil-mycophenolate (2-3 g daily), and prednisolone (tapering regimen, 0.15 mg/kg). Concomitant medication included pantozol, amlodipine, ramipril, magnesium, allopurinol, torasemide, fluvastatine, and Ca-D3. Two serologic CMV replications were treated with ganciclovir i.v. The cardiovascular risk profile prior to transplantation included tobacco use, arterial hypertension, and male gender.

During a routine follow-up visit in March 2013 leukocytosis of 33.000/*μ*L, thrombocytosis of 655.000/*μ*L, and anemia of 11.2 g/dL were noted. The differential blood count was myelocytes 6%, metamyelocytes 2%, bands 5%, segmented granulocytes 75%, eosinophils 2%, basophils 2%, monocytes 5%, and lymphocytes 3%. Accordingly, a bone marrow analysis was done that revealed elevation of megakaryocytes and a disbalance between granulopoiesis and erythropoiesis of 4 : 1 but no elevation of blasts or promyelocytes. Conventional cytogenetics analysis demonstrated the typical t(9;22)(q34;q11) translocation in all analysed nuclei and a multiplex PCR confirmed the presence of BCR-ABL transcripts establishing the diagnosis of chronic phase chronic myeloid leukemia. No hepatosplenomegaly was present allocating this patient into the low-risk category by EUTOS-score [28].

As there are currently three tyrosine kinase inhibitors (imatinib, nilotinib, and dasatinib) with approval for the first-line treatment of CML, the choice of therapy was primarily made on the basis of the individual tolerability profile. As nilotinib is associated with a cardiovascular risk and dasatinib is linked to pleural and pericardial effusion and pulmonary artery hypertension, imatinib 400 mg was chosen [32, 33].

Imatinib was well tolerated and within 3 months normalisation of peripheral blood cell counts, compatible with a complete hematologic response, was observed. At the time of CML diagnosis maintenance immunosuppression was switched to a double regimen (cyclosporine A trough level 100–120 ng/mL and prednisolone 0.15 mg/kg). A bone marrow analysis taken 15 months after start of imatinib showed a complete cytogenetic remission. However, in 10 out of 25 otherwise normal analysed metaphases a loss of the y chromosome was detected. So far, imatinib is well tolerated in this patient without any drug related adverse events.

Echocardiographic examinations done at diagnosis of CML and 14 months after initiation of imatinib documented a left ventricular ejection fraction of at least 60% indicating no cardiotoxicity of tyrosine kinase inhibitor therapy.

## 3. Discussion

The development of hematologic malignancies is a rare clinical complication in the post- organ-transplant population and most frequently occurs as Non-Hodgkin-Lymphoma (PTLD).

Since the first description by Battin et al. (1976) altogether 34 patients developing CML after SOT were reported, primarily as case reports, and 15 of these cases were published within the past 5 years, indicating a growing interest in this phenomenon [4–27]. In a most recent report the frequency of CML developing in SOT patients was calculated relating the total transplant numbers in a single centre and the number of CML cases observed in this group of patients to the age- and gender-matched US population [26]. Despite the low denominator of 3089 individuals in this study an age adjusted incidence rate of 34.7 per 100 000 was observed that significantly (*P* < 0.0001) exceeds the US incidence of 1.6 per 100 000 [26]. Thus, under the assumption that SOT carries an elevated risk of CML, these numbers will eventually increase in the future as both the total number of transplants and the age of organ recipients are increasing.

Generally, two types of mechanisms must be differentiated in the clinical setting of a de-novo posttransplant malignancy: (1) the unfrequent cases of preexisting malignancies that are transferred with the donor organ and (2) malignancies manifesting after initiation of immunosuppressive therapy. In the first case HLA-typing or molecular clonal testing of neoplastic tissue can be applied to rule out donor derived neoplasms while in the latter case the exact pathomechanism is not understood but impaired immune surveillance together with intrinsic mutagenic potential may play a significant role. But also the effect of either infectious (viral) or toxic (immune suppressive drugs) factors could contribute as an extrinsic cause of transformation. In this regard the observation of DNA lesions caused by azathioprine in combination with UVA radiation is of note [34]. Still, the exact role of the immunosuppressive drugs in the evolution of posttransplantation malignancies needs to be elucidated, but it is of interest that these cases continue to appear while azathioprine is being more and more replaced by alternative drugs such as mycophenolate mofetil and tacrolimus.

Several aspects in the setting of CML developing after SOT are of note.

With the various tyrosine kinase inhibitors that are currently available for the first-line treatment the prognosis of CML has improved dramatically over the past 15 years [28]. To what extent the specific clinical setting of patients with CML following SOT may impact response is unclear. Certainly, the underlying disease leading to transplantation, potential drug interactions, and a potentially low clinical performance status could negatively impact response and survival rates in individual patients. On the other hand due to the more frequent visits of patients following SOT patients developing CML may eventually be recognised earlier and already at a lower risk. This hypothesis is reflected by the observation that in our patient as well as in many of the currently published cases WBC were relatively low (<35.000 per *μ*L) when CML was diagnosed (Table 1).

Probably because of relative short follow-up intervals in none of the reports presented here deep molecular responses that would allow drug discontinuations were reported. However, as immunological mechanisms are currently discussed to be involved in patients stopping TKI therapy after long-term molecular remission, we hypothesize that in patients with CML following immunosuppression fewer patients would remain BCR-ABL negative after discontinuation as long as immunosuppression is continued.

To our knowledge we here describe the fourth patient with CML following heart transplantation and the third case of this kind that was treated with a tyrosine kinase inhibitor [10, 24]. But other than in the cases reported by Sbenghe et al. and Menon et al. who received tacrolimus in addition to mofetil-mycophenolate in our case cyclosporine was given [24, 27]. However, the significance of the various immunosuppressive drugs given in these patients is still unclear. The case reported by Menon et al. is of specific interest as it describes a secondary CML developing after acute myeloid leukemia [27]. In this pediatric case heart transplantation was carried out because of anthracycline-induced cardiomyopathy [27]. With the exception of a past case by Frist et al. all other cases of CML following heart transplantation are characterized by a relatively short latency of 9 to 16 months [10, 24, 27]. This observation could be associated to the relatively dose intensive immunosuppressive regimens used in heart transplantation.

Most of the published cases were treated with imatinib as first-line TKI. In general, all three currently approved first-line options (imatinib, nilotinib, and dasatinib) can be chosen as well in SOT patients. However, as the toxicity profiles of the second generation TKIs dasatinib and nilotinib include immunosuppression, atherosclerotic events, pulmonary artery hypertension, and pleural effusion, an initial trial with imatinib, despite its less favourable response rates, may be the preferred treatment option in many of these patients [32, 33]. As in our patient numerous cardiovascular risk factors were present and already a major cardiac intervention was performed, we also preferred imatinib over a second generation TKI as first-line option.

In summary, CML following immunosuppressive therapy beyond its clinical implications is an interesting model of immune surveillance in tumor biology. As there are only few cases of Ph1 chromosome negative chronic myeloproliferative neoplasms arising after immunosuppression, we suspect this phenomenon to be specifically linked to CML. We believe that both preclinical and clinical investigations that address the specific mechanisms of immunosuppressive drugs and the acquisition of the t(9;22)(q34;q11) could be of interest.

## Figures and Tables

**Table 1:** Currently published case reports of CML developing after solid organ transplantation.

Reference	Age (years)	Sex	Immunosuppression	Latency (mo)	Best response to TKI	Time to best response (mo)	WBC/*μ*L	Comment
*Cases of kidney transplantation *								
Battin et al., 1976 [4]	15	m	A	48	—	—	—	
Adler et al., 1978 [5]	18	m	A, S, R	32	—	—	—	
Lubynski et al., 1978 [6]	20	m	A, S	35	—	—	69.000	
Mooy et al., 1978 [7]	40	m	A, S, Actinomycin C	48	—	—	—	
Hilario et al., 1980 [8]	26	f	A, S	60	—	—	—	
Kirchner et al., 1983 [9]	31	f	A, S, R	42	—	—	73.000	Retransplantation
Sanz et al., 1996 [11]	59	m	A	72	—	—	—	
Stein et al., 1978 [12]	19	m	A, S	18	—	—	—	
Mignozzi and Picca, 2001 [13]	16	m	A, OKT-3, C, S	96	—	—	65.000	
Pescovitz et al., 1996 [14]	52	m	A, ALG, S, C	0	—	—	14.700	
Penn, 1998 [15]	Child	—	A, OKT-3	—	—	—	—	
Pelloso et al., 2005 [16]	37	m	A, C, S	30	—	—	92.000	
Koca et al., 2005 [17]	33	m	A, S, C	87	Imatinib/CCyR	—	—	
Thierry et al., 2007 [19]	53	f	ATG, C, M, S	25	Imatinib/MR4	8	28.600	
Fontana et al., 2008 [20]	54	f	T, S, M	63	Imatinib/CHR	2	87.540	
le Coutre et al., 2010 [21]	39	m	T, C	38	Imatinib/NA	NA	41.300	
le Coutre et al., 2010 [21]	47	f	T, C, S	183	Nilotinib/CCyR	NA	27.700	Retransplantation
le Coutre et al., 2010 [21]	54	f	C, M, S	11	NA	NA	139.000	
Castillo-Rama et al., 2009 [22]	48	m	A, S, C, M	204	Imatinib/MR4	60	34.500	Retransplantation
Sayar et al., 2011 [23]	18	m	ATG, S, M, T	13	Imatinib/HR	0,5	37.000	
Sayar et al., 2011 [23]	56	m	ATG, S, M, T	10	Imatinib/HR	NA	39.000	
Oskuei et al., 2014 [25]	63	m	C, M, S	26	Imatinib/CHR	2	59.000	
Dhanarajan et al., 2014 [26]	59	m	Sirolimus	120	Imatinib/CCyR	6	NA	
Dhanarajan et al., 2014 [26]	77	f	S, T, M	12	Imatinib/HR	1	NA	
Dhanarajan et al., 2014 [26]	48	f	S, T	72	Imatinib/CCyR	6	NA	Combined kidney pancreas transplantation
*Cases of liver transplantation *								
Fontana et al., 2008 [20]	55	m	T, S	64	Imatinib/CHR	3	6.200	
Fontana et al., 2008 [20]	59	f	T, S	ca. 18	Imatinib/CHR	6	84.600	
le Coutre et al., 2010 [21]	63	m	T	32	Imatinib/CCyR	4	37.700	
Dhanarajan et al., 2014 [26]	52	m	S, T	48	Imatinib/MR4	18	NA	
*Cases of lung transplantation *								
Dhanarajan et al., 2014 [26]	50	m	S, T, A	18	Imatinib/MR4	22	NA	
*Cases of heart transplantation *								
Frist and Biggs. 1994 [10]	31	m	A, C, OKT-3, S, R	84	—	—	—	Retransplantation
Sbenghe et al., 2011 [24]	54	m	T, M	14	Imatinib/NA	NA	68.000	
Menon et al., 2014 [27]	4	f	T, M	9	Dasatinib/NA	NA	77.700	Secondary CML after AML
Present case	63	m	C, M, S	16	Imatinib/CCyR	15	33.000	

A, azathioprine; C, cyclosporine A; S, steroids; ATG/ALG, antithymocyte globulin/antilymphocyte globulin; M, mycophenolate mofetil; T, tacrolimus; R, radiation; NA, not available; WBC: white blood cell count at diagnosis. Retransplantation: several transplantations of the same organ were performed sequentially. Modified according to le Coutre et al. [21].
